# There is no right time for accountability for women’s, children’s and adolescents’ health

**DOI:** 10.1080/16549716.2022.2067399

**Published:** 2022-09-13

**Authors:** Agbessi Amouzou, Neff Walker

**Affiliations:** Department of International Health, Bloomberg School of Public Health, Johns Hopkins University, Baltimore, Maryland, USAaamouzo1@jhu.edu

## Accounting for results in RMNCH&N

Accounting for progress in reproductive, maternal, newborn and child health and nutrition (RMNCH&N) is a continuing endeavor and a key determinant for progress. Accountability requires adequate investment, appropriate implementation, and rigorous assessment of results along a well-defined logic framework. In May 2014 at the ‘*Saving Every Woman Every Child: Within Arm’s Reach*’ summit, as the period of the Millennium Development Goals was ending and discussions were under way for the current development agenda, the Canadian government committed 3.5 billion dollars to improving global maternal, newborn and child health, a follow-on to its five-year Muskoka initiative [1]. Ensuring real accountability for this large investment necessitated development of plans for effective implementation and a rigorous and comprehensive evaluation framework. Donors and development partners have traditionally measured progress in the equitable coverage and quality of RMNCH&N interventions and their mortality impact at national and global levels using existing national survey programs such as the demographic and health surveys (DHS), the multiple indicator cluster surveys (MICS), service provisional assessments (SPA), and service availability and readiness assessments (SARA). However, progress at national level is determined by effective implementation of RMNCH&N interventions and strategies, often at subnational levels, undertaken by governments and their non-governmental agency (NGO) partners. The existing tools are insufficiently flexible in their timing and scope for use by these partners for interim assessments of subnational progress and the effectiveness of the deployed programs and strategies. Furthermore, the evaluation of the effectiveness of large-scale government programs demands an approach that differs from that used in controlled trials. These programs often rely on interventions already proven efficacious in controlled trials, and the critical question therefore becomes whether they are effective when deployed to large populations. Their evaluation must ensure that the program is properly designed and packaged based on evidence, is implemented with enough strength and quality to generate impact, and is resulting in expected gains in coverage and impact on mortality. Because these programs are already deployed at large scale, a lack of effectiveness is detrimental to the population served, results in a waste of limited resources, and undermines continued funding. Accountability for positive gains lies with all stakeholders, including donors, implementers, and evaluators. Large scale effectiveness evaluations must therefore utilize a suite of tools and approaches that support strong program strategies implemented with evidence of measurable positive impact. To address this gap, the Canadian government funded the Real Accountability, Data Analysis for Results project (RADAR) in 2016 to develop a holistic evaluation framework and suite of tools that support rigorous accountability of resources invested by Canada through its supported NGOs and country governments. A second objective of RADAR is to build the capacity of Canadian NGOs in the use of these tools.

This Special Issue brings together a set of articles describing the RADAR methodological approach, the resulting tools, and experiences in their implementation. The individual tools build on existing, validated tools that are often used individually, and do not require further validation in terms of the accuracy of their measures beyond feasibility testing. Their adaptation consisted of ensuring rapid implementation using streamlined and adaptable instruments with accompanying digital solutions for rapid data capture, monitoring and analysis, flexibility, and feasibility for use by NGOs with limited resources.

## Four linked tools for real accountability, data analysis for results

Effectiveness evaluation processes must be demystified using simple terminologies, questions and approaches that are understandable by program implementers and potential evaluators. The effectiveness evaluation framework and the need for the RADAR tools is described by Amouzou and colleagues, who also highlight the broader context and challenges for effectiveness evaluation of large-scale programs [2]. The RADAR framework defines interlinked and stepwise priority questions to be addressed at each stage of the evaluation. Five priority questions are defined, each leading to a measurement tool to address the question. Four tools are presented, each in a separate paper. A supportive effectiveness evaluation does not jump to assessing whether the program contributed to mortality and nutrition impact. Instead, evaluators must first clarify, at inception, whether the program was appropriately designed in the implementation context and focuses on delivery of relevant high impact interventions. The Lives Saved Tool (*LiST*), described by Walker and Tam, is the first tool and offers a rigorous approach to addressing this question [3]. The second priority question, which leads to the second tool, asks about the integrity of the proposed pathway through which program inputs, processes and outputs are expected to lead to coverage increases and reductions in mortality. An evidence-based and clearly specified impact model is the backbone of a sound evaluation. Roberton and Sawadogo-Lewis present an approach and digital tool for developing a sound impact model [4]. Answering these two fundamental questions lays the groundwork for answering the third question on whether the program is strongly implemented, with attention to quality of care and utilization. Ideally, program implementation strength and quality of care would be measured through routine program data collected as part of the monitoring and evaluation framework of the program. However, systematic documentation of the program and collection of indicators to measure program strength and quality of care are rarely part of routine monitoring by program implementers, forcing evaluators to design special tools to capture these aspects of the program. The third tool aims to assess the program implementation strength and quality of care. Measuring quality of care can be complex, especially because standard definitions of quality are available for few RMNCH&N interventions. Marx and colleagues introduce tested tools for measuring quality of care [5]. Well-designed, strongly implemented programs should lead to increased levels of intervention coverage in target populations. The fourth tool is a comprehensive tool for measuring the coverage of interventions and is described by Munos and colleagues. The tool includes the survey questionnaire built on open data kits and accompanying routines for sampling, mapping and calculating sample size [6]. The final priority evaluation question is whether the program had an impact. Impact can be expressed in terms of reduced mortality, fertility, morbidity, or nutritional status. Given the large sample sizes and complexities required for measuring changes in mortality and fertility, the RADAR suite of tools does not include dedicated tools measuring these outcomes. However, *LiST* has been validated and is used widely to model impact on mortality and nutritional status using changes in coverage of interventions [3]. Ideally, a comprehensive effectiveness evaluation would also incorporate an assessment of inequities by relevant equity stratifiers such as socio-economic status, gender, and age. Morgan and colleagues discuss an approach for incorporating gender inequity assessments within the coverage tool and analysis [7].

## Examples of application of the tools

While the RADAR tools are conceived as a package for a full effectiveness evaluation, they can also be used individually to support the assessment of specific aspects of a program. Luay and colleagues report on using the implementation strength and quality of care tool to assess the strength and quality of integrated community case management of childhood illness in the Mali [8]. They demonstrated the usefulness of the tool in validating program monitoring and supervision data and generating further results to strengthen the program.

## A word of caution

The RADAR tools are not designed to supplant the need for inherent and rigorous routine monitoring and evaluation of programs implemented at scale, including the use of routine health information system data. Neither do they replace the need for continuous engagement at local level to understand the bottlenecks and impediments to successful program implementation. The RADAR tools support these other essential processes.

Success in meeting the need for sound effectiveness evaluations requires leadership and ownership from concerned governments and their local partners, committed to improving the quality, sustainability and scalability of their programs. Accountability assessment is not a one-off activity. It must be a continuous process dedicated to achieving and maintaining universal health coverage. The time for accountability is always right!
